# Investigation of the DNA damage response to SFOM-0046, a new small-molecule drug inducing DNA double-strand breaks

**DOI:** 10.1038/srep23302

**Published:** 2016-03-22

**Authors:** Joris Pauty, Marie-France Côté, Amélie Rodrigue, Denis Velic, Jean-Yves Masson, Sébastien Fortin

**Affiliations:** 1 Genome Stability Laboratory, CHU de Québec Research Center, Oncology Axis, Hôtel-Dieu-de-Québec, 9 McMahon, Quebec City, QC, G1R 2J6, Canada; 2 Department of Molecular Biology, Medical Biochemistry and Pathology, Faculty of Medicine, Laval University, Quebec City, QC, G1V 0A6, Canada; 3 CHU de Québec Research Center, Oncology Axis, Hôpital Saint-François d’Assise, 10 de l’Espinay, Quebec City, QC, G1L 3L5, Canada; 4 Faculty of Pharmacy, Laval University, Quebec City, QC, G1V 0A6, Canada

## Abstract

2-Ethylphenyl 4-(3-ethylureido)benzenesulfonate (SFOM-0046) is a novel anticancer agent that arrests cell cycle in S-phase and causes DNA replication stress leading to the phosphorylation of H2AX into γ-H2AX. First, using the M21, HT29, HT-1080 and HeLa cell lines, we confirmed that S-phase cell cycle arrest and γ-H2AX foci induction by SFOM-0046 is a general mechanism occurring in diverse cancer cell lines. In addition to γ-H2AX, SFOM-0046 activates preferentially ATR-Chk1 in M21 and HT29 cells while both ATR-Chk1 and ATM-Chk2 pathways are activated in HCT116 cells. Co-localization of SFOM-0046-induced 53BP1 foci with γ-H2AX foci validates that the DNA damage generated corresponds to double-strand-breaks (DSBs). Consistent with an S-phase arrest, SFOM-0046 treatment induces RAD51 foci formation but not DNA-PKcs foci, confirming that homologous recombination is the major DSB repair pathway targeted by the drug. Furthermore, using isogenic HCT116 p53+/+ and HCT116 p53−/− cells, we showed that p53 plays a key role in the survival mechanism to SFOM-0046. Finally, SFOM-0046 exhibits a dose-dependent antitumor activity on human fibrosarcoma HT-1080 tumours grafted onto chick chorioallantoic membranes without showing embryo toxicity even at high doses. Altogether, our results highlight SFOM-0046 as a very promising drug that induces a replication stress response.

The DNA damage response has a crucial and natural function to maintain the genome integrity in all eukaryotic cells^1^. It comprises a complex network of signalling and transduction pathways involving proteins that sense DNA damage and coordinate many cellular processes, including DNA repair, damage tolerance, transcriptional responses, DNA damage checkpoints and apoptosis^2^. Ataxia telangiectasia mutated (ATM) and Rad3-related (ATR) kinases are members of the PI-3 family of serine-threonine kinases. Both proteins are playing key roles in the DNA damage response by bridging signals from the damage sensors to the signalling and repair pathways^3^. Activation of ATM or/and ATR kinases leads to the phosphorylation of downstream effectors including Chk2 for ATM (ATM-Chk2 pathway), Chk1 for ATR (ATR-Chk1 pathway) and histone H2AX (γ-H2AX)^4^,^5^. Chk1 and Chk2 are key cell cycle checkpoint kinases while histone H2AX is crucial for recruiting and maintaining downstream effectors and repair proteins at DNA damage sites. Notably, the phosphorylation of H2AX into γ-H2AX, a proper indicator of DNA damage and replication stress, is considered a hallmark of the number of DNA double-strand breaks (DSBs) generated^6^. Although ATM and ATR partially play overlapping, additive and cooperative roles in DNA damage response, they play also distinct roles during DNA repair^7^. ATM is mostly responsible to respond to DNA DSBs as well as disruption of the chromatin structure while ATR responds primarily to single-stranded DNA induced by UV damage and stalled replication forks^8^. Another important player of the DNA damage response is 53BP1, which binds damaged chromatin through multiple histone modifications initiated by MDC1^9^.

There are two main pathways to repair DNA DSBs in eukaryotic cells, homologous recombination (HR) and non-homologous end joining (NHEJ)^10^. The initial step in NHEJ is the recognition and binding of the Ku heterodimer, composed of the Ku70 and Ku80, proteins to the DSB^11^. The Ku heterodimer then recruits, either directly or indirectly, DNA-PKcs, DNA ligase IV, XRCC4, XRCC4-like factor (XLF), and aprataxin-and-PNK-like factor (APLF) to DSBs^12^. DNA-PKcs is autophosphorylated at Thr2609 *in vivo* in a Ku-dependent manner in response to ionizing radiation^13^. If the ends of the DSBs are compatible and exhibit 3′ hydroxyl and 5′ phosphate termini, end processing by the Artemis nuclease is not necessary^14^. The DNA ligase IV complex, consisting of the catalytic subunit DNA ligase IV and its cofactor XRCC4, performs the ligation step of the ends to complete DNA repair of the DSBs^15^.

The NHEJ repair mechanism occurs throughout the cell cycle and may introduce mutations at repair sites while the HR mechanism is considered to be error free. HR can be divided into presynaptic, synaptic, and post-synaptic stages. In the presynaptic phase, HR is initiated by the binding of the heterotrimeric MRE11-RAD50-NBS1 (MRN) complex to the broken DNA ends^16^. MRE11 initiates 5′-3′ nucleolytic processing, which is continued by the combined action of EXO1, BLM, and DNA2. Next, the heterotrimeric ssDNA-binding protein replication protein A (RPA) coats the resected DNA and inhibits secondary structures formation to facilitate the loading of RAD51^17^, a step that is mediated by BRCA2 and/or PALB2^18^. In the synaptic phase, RAD51 promotes DNA strand exchange between the broken and the targeted homologous DNA to form the displacement loop (D-loop), which contains the novel heteroduplex DNA and the displaced strand of the donor DNA^19^. In the postsynaptic phase, DNA synthesis is primed from the broken 3′ end. Mitotic DSBs are preferentially repaired by synthesis-dependent strand annealing (SDSA), in which the invading strand is displaced after DNA synthesis and then anneals with the second DSB end^20^.

We recently uncovered a new class of compounds designated as *N*-phenyl ureidobenzenesulfonates (PUB-SOs, Fig. 1) that exhibited antiproliferative activity in the micromolar range on several human tumour cell lines notably M21 skin melanoma, estrogen-dependent MCF-7 breast adenocarcinoma and HT29 colon adenocarcinoma^21^,^22^. PUB-SOs blocked Jurkat cell cycle progression in S-phase and induced the formation of γ-H2AX in M21 cells, indicating that these new agents led to DNA replication stress. The screening of our PUB-SOs chemolibrary identified 2-ethylphenyl 4-(3-ethylureido)benzenesulfonate (SFOM-0046) as a promising new anticancer drug. Interestingly, the molecular structure of SFOM-0046 is devoid of elements allowing spontaneous DNA alkylation or DNA intercalation. Consequently, its mechanism of action is not related to a direct interaction with DNA to generate replication stress. In this study, we investigated the DNA-damaging properties of SFOM-0046 and its effect on the DNA damage response using M21 and HT29 cells. We also analysed the antitumoral activity of SFOM-0046 on the HT-1080 fibrosarcoma cell line grafted onto the chick chorioallantoic membranes (CAM assay).

## Results

### S-phase arrest and induction of γ-H2AX by SFOM-0046 is a general mechanism of action

**Figure 2:**
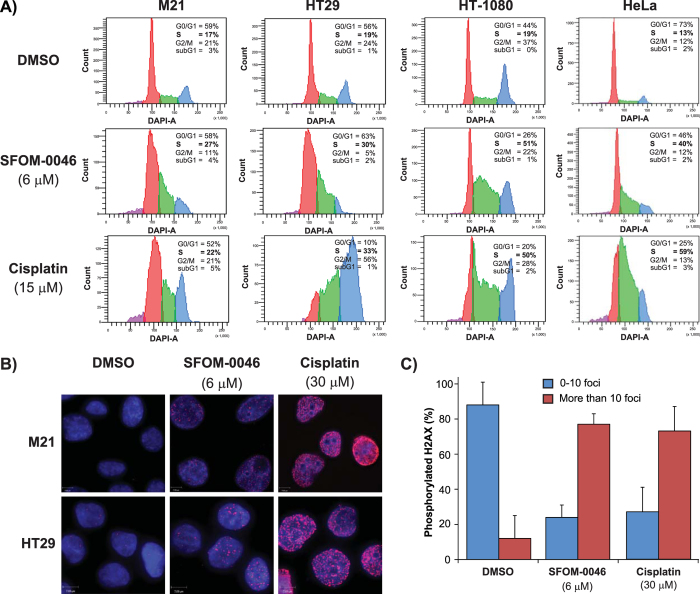
(**A**) Effect of a 24-h treatment with DMSO, SFOM-0046 (6 μM) or cisplatin (15 μM) on the cell cycle progression of M21, HT29, HT-1080 and HeLa cells. (**B**) Effect of DMSO, SFOM-0046 (6 μM) and cisplatin (30 μM) on the phosphorylation of H2AX into γ-H2AX after 24 h of treatment in M21 and HT29 cells. (**C**) Quantification of the number of M21 cells displaying less and more than 10 γ-H2AX foci.

To evidence that S-phase cell cycle arrest by SFOM-0046 is part of a general mechanism of action that is not specific to Jurkat cells^22^, we have investigated the effect of SFOM-0046 on cell cycle progression of human M21 melanoma, HT29 colon adenocarcinoma, HT-1080 fibrosarcoma, and HeLa cervix adenocarcinoma cells using flow cytometry. Cells were treated with DMSO, cisplatin (15 μM, as a positive control) or SFOM-0046 (6 μM) for 24 h prior to flow cytometry analysis (Fig. 2A). When treated with DMSO, the majority of the cells were in G0/G1-phase. Indeed, 59, 56, 44 and 73% were in G0/G1-phase for M21, HT29, HT-1080 and HeLa cells, respectively. Moreover, fewer cells were in S-phase with 17, 19, 19 and 13% of cells for M21, HT29, HT-1080 and HeLa cells, respectively. Treatment with cisplatin, a DNA-interstrand and -intrastrand crosslinker that interferes with DNA replication^23^, resulted in an S-phase accumulation of 22, 33, 50 and 59% for M21, HT29, HT-1080 and HeLa cells, respectively. These results are similar to those obtained with M21, HT29, HT-1080 and HeLa cells treated with 6 μM of SFOM-0046, where 27, 30, 51 and 40% of the cells were accumulated in S-phase, respectively. Thereafter, we set out to confirm our previous results with M21 cells showing that SFOM-0046-induced S-phase arrest is due to DNA damage accumulation, using another cell line (HT29). To that end, we performed immunofluorescence staining to detect the phosphorylation of the histone variant H2AX, known to be phosphorylated on Ser139^24^,^25^ to form γ-H2AX foci in response to DNA DSBs. M21 and HT29 cells were treated either with SFOM-0046 (6 μM), cisplatin (30 μM) or DMSO for 24 h, followed by immunofluorescence staining to detect the formation of γ-H2AX foci. As depicted in Fig. 2B, SFOM-0046 and the positive control cisplatin induced an increase in the number of γ-H2AX foci compared to DMSO, used as excipient and negative control, in both cell lines. Quantification revealed that upon treatment with SFOM-0046 or cisplatin at least 70% of M21 cells had 10 or more γ-H2AX foci compared to only 10% with DMSO treatment. These results were also confirmed in all other cancer cell lines tested so far, including HeLa and HCT116. Altogether, these results indicate that the accumulation of cells in S-phase and the generation of DNA replication stress induced by SFOM-0046 is not cell-type specific, supporting a general mechanism of action in cancer cell lines.

### SFOM-0046 activates ATR-Chk1 and ATM-Chk2 pathways

**Figure 3:**
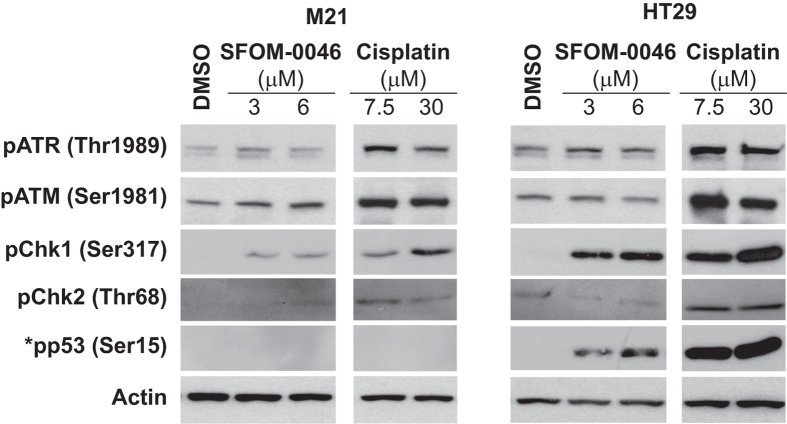
Dose-dependent activation of DNA damage response by the ATR-Chk1 and ATM-Chk2 pathways following a 24-h treatment with SFOM-0046 or cisplatin in M21 and HT29 cells. *Expression of p53 in M21 cells is deficient comparatively to HT29 cells (Fig. 6C). The gels/blots have been run under the same experimental conditions. Full-length western blots are included in the supplementary information (Fig. S1).

The latter results suggested that SFOM-0046 induces DNA replication stress by elevating DNA damage, causing an arrest of cell cycle progression in S-phase. To identify which DNA damage checkpoint is involved in promoting the S-phase arrest, M21 and HT29 cells were treated with DMSO or escalating concentrations of SFOM-0046 or cisplatin for 24 h. Whole cell extracts were then prepared and analysed by immunoblotting to detect the phosphorylated, or activated, forms of ATR, ATM, Chk1, Chk2 and p53. As shown in Fig. 3, HT29 and M21 cells exhibited higher levels of phosphorylation of ATR (Thr1989), ATM (Ser1981), Chk1 (Ser317) and Chk2 (Thr68) after a 24-h cisplatin treatment. Phosphorylation of ATR, ATM, and Chk1 was also detected with SFOM-0046 treatment. However, Chk2 was phosphorylated to a much lesser extent than Chk1 in both cell lines, suggesting that ATM is activated more weakly than ATR by SFOM-0046. In support of this, the kinetics of activation of ATR and Chk1 by SFOM-0046 (6 μM) at 2, 4, 6, 16, and 24 h shows that ATR is quickly phosphorylated (2 h) followed by activation of Chk1 at 6 h (Fig. S2). These results suggest that SFOM-0046 induces preferentially the activation of ATR and also that the activation of Chk1 and Chk2 is mainly mediated by respective phosphorylation of ATR and ATM. While the G2/M checkpoint uses, at least in part, both pathways to activate and maintain cell cycle arrest, replication checkpoints preferentially activate the ATR-Chk1 pathway^26^. The rapid and specific activation of ATR and Chk1 at the early time points supports this finding. However, a sustained replication-stress signalling could account for the observed weak activation of ATM. The phosphorylation of p53 was also assessed in our cells lines. M21 appeared to be defective in p53 activation as no phosphorylation of p53 was observed. Conversely, p53 was highly phosphorylated in HT29 following SFOM-0046.

### The replication stress induced by SFOM-0046 is DNA DSBs

**Figure 4:**
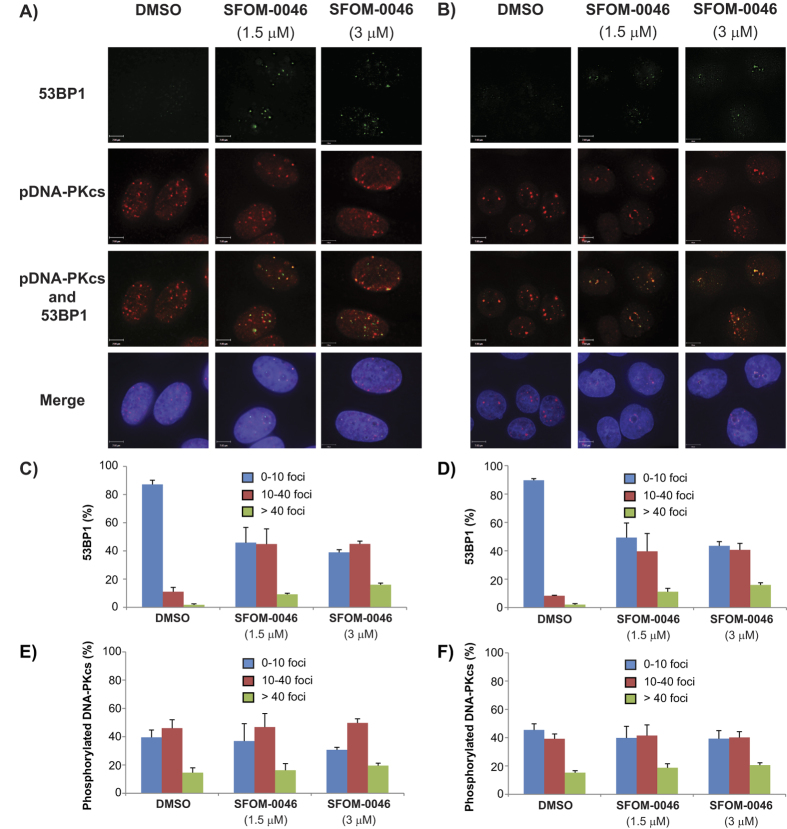
Effect of DMSO and SFOM-0046 at 1.5 and 3 μM on the phosphorylation of 53BP1 and DNA-PKcs after 24 h of treatment on: (**A**) M21 and (**B**) HT29 cells. Quantification of the number of : (**C**) M21 and (**D**) HT29 cells displaying less than 10, 10–40 and over 40 53BP1 foci. Quantification of the number of: (**E**) M21 and (**F**) HT29 cells displaying less than 10, 10–40 and over 40 DNA-PK foci.

The latter results suggested that SFOM-0046 induces replication stress and the detection of γ-H2AX foci indicated that DNA damage, most likely DSBs, could be the cause of this replication stress. To further validate the nature of the damage induced by SFOM-0046, we performed immunofluorescence staining to detect 53BP1 foci, a second marker of DNA DSBs^27^. M21 and HT29 cells were treated either with SFOM-0046 (1.5 and 3 μM) or DMSO for 24 h, followed by immunofluorescence staining for 53BP1 foci. As shown in Fig. 4, DMSO treatment did not induce 53BP1 foci while both SFOM-0046 treatments (1.5 and 3 μM) induced significantly 53BP1 in the two cell lines. These results confirm that replication stresses induced by SFOM-0046 are DNA DSBs.

### Treatment with SFOM-0046 activates homologous recombination repair

**Figure 5:**
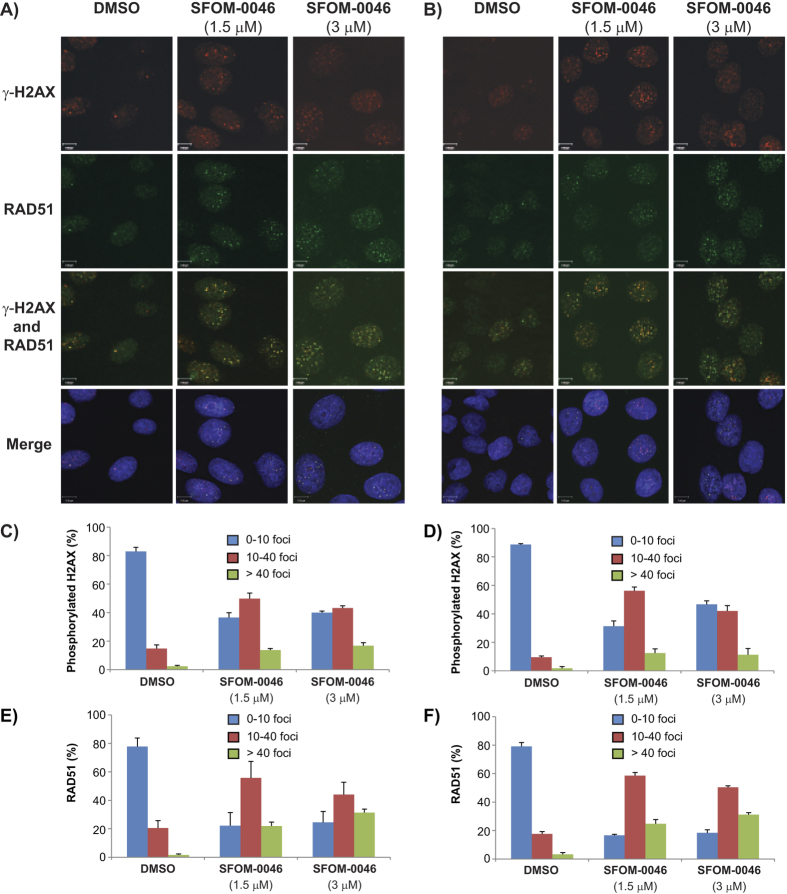
Effect of DMSO and SFOM-0046 at 1.5 and 3 μM on the phosphorylation of H2AX and RAD51 after 24 h of treatment on: (**A**) M21 and (**B**) HT29 cells. Quantification of the number on: (**C**) M21 and (**D**) HT29 cells displaying less than 10, 10–40 and over 40 RAD51 foci.

Since treatment with SFOM-0046 results in accumulation of cells in S-phase, we expected the induced DNA DSBs would be repaired by the HR pathway instead of NHEJ. Indeed, HR is more prone to repair DSBs in S-phase while NHEJ pathway is known to be activated throughout the cell cycle and favoured in G1-phase^28^. To that end, we performed immunofluorescence staining to detect RAD51 and phosphorylated DNA-PKcs, two essential kinases involved in the HR and NHEJ cascades, respectively^28^,^29^. Hence, M21 and HT29 cells were treated with the excipient (DMSO) or with 1.5 or 3 μM of SFOM-0046 for 24 h followed by immunofluorescence staining of Thr2609-phospho-DNA-PKcs, γ-H2AX and RAD51. As expected, treatments with SFOM-0046 (1.5 and 3 μM) did not induce the formation of phosphorylated DNA-PKcs foci when compared to the excipient (Fig. 4). In contrast, the number of γ-H2AX and RAD51 foci generated by SFOM-0046 in M21 and HT29 cells was concentration-dependent (Fig. 5). In addition, RAD51 foci co-localized with γ-H2AX foci. Our results confirm that the HR pathway is activated following DNA damage caused by SFOM-0046.

### p53-deficient cells are more sensitive to SFOM-0046

**Figure 6:**
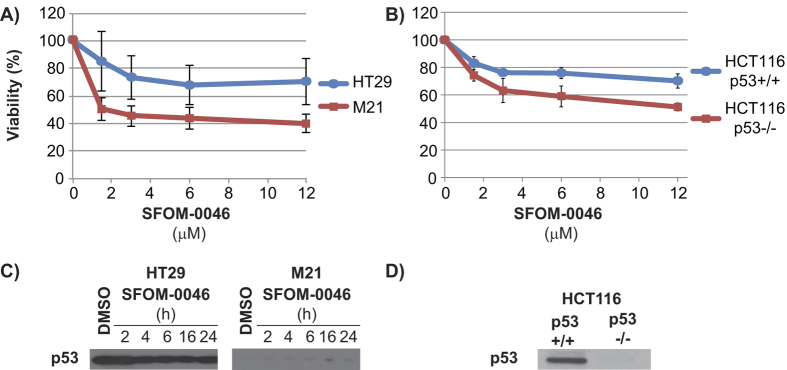
Cells viability of (**A**) HT29 and M21 as well as (**B**) HCT116 (p53+/+ and p53−/−) cells treated with escalating concentrations of SFOM-0046. (**C**) Effect of DMSO and SFOM-0046 on the p53 expression level of HT29 and M21 cells at different treatment times. (**D**) Confirmation of the p53 (p en minuscule) status of the isogenic HCT116 p53+/+ and HCT116 p53−/− cells.

Our previous studies showed that M21 cells are more sensitive to PUB-SOs than HT29 cells^22^. This result was also confirmed by a cell viability assay performed after 96 h of treatment with increasing concentrations of SFOM-0046 (Fig. 6A). Moreover, studies on the phosphorylation of p53 in DNA damage response show that p53 is not phosphorylated in M21 cells as compared to HT29 cells. In addition, studies report that M21 cells are expressing a functional p53 with a G266E mutation^30^,^31^. Nevertheless, the quantification of p53 proteins in M21 and HT29 cell lines shows that p53 is very weakly expressed in M21 cells comparatively to HT29 cells (Fig. 6C). This result suggests that p53 plays a key role in the cell survival after treatment with SFOM-0046. To support this hypothesis, we studied the effect of SFOM-0046 on isogenic HCT116 cell lines expressing (p53+/+) or not p53 (p53−/−). First, the isogenic HCT116 p53 +/+ and p53 −/− status were confirmed by the detection of the p53 protein by immunoblot (Fig. 6D). Then, the cell viability assay after 96 h of treatment with increasing concentration of SFOM-0046 showed that HCT116 p53−/− cells are more sensitive to SFOM-0046 than p53+/+ cells (Fig. 6B). These results evidence that the expression of p53 plays a role in the cell survival to SFOM-0046 treatment.

### SFOM-0046 induces a general mechanism of DNA damage response

**Figure 7:**
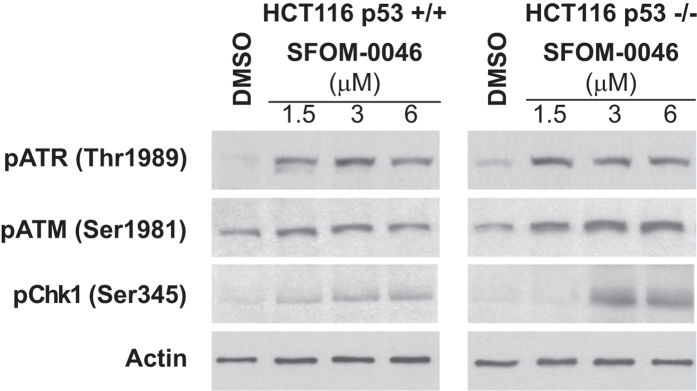
Dose-dependent phosphorylation of ATR and ATM in response to SFOM-0046 (1.5, 3 and 6 μM) in isogenic HCT116 p53+/+ and HCT116 p53−/− cells.

**Figure 8:**
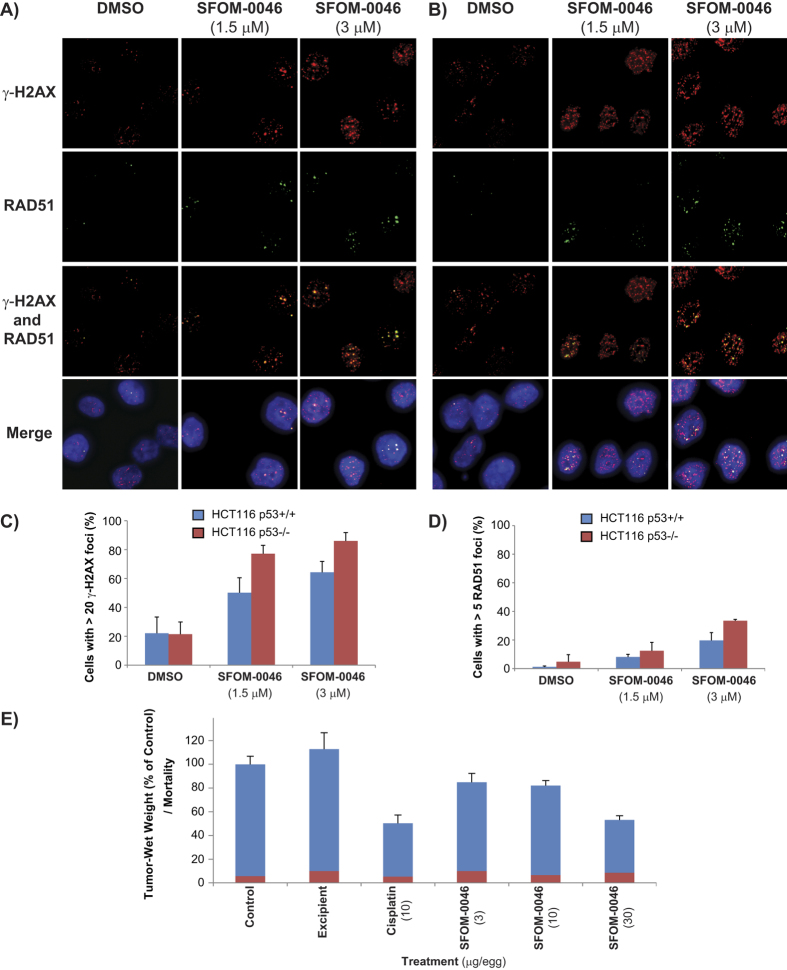
Effect of DMSO and SFOM-0046 at 1.5 and 3 μM on the phosphorylation of H2AX and RAD51 after 24 h of treatment in isogenic HCT116 (**A**) p53+/+ and (**B**) p53 −/− cells. Quantification of the number of cells with: (**C**) more than 20 γ-H2AX foci and (**D**) over 5 RAD51 foci. (**E**) Effect of SFOM-0046 and cisplatin on the growth of HT-1080 tumours and their toxicity on chick embryos in the CAM assay. Blue bars represent the percentage of wet-weight of tumours treated with or without the excipient. Red bars represent the percentage of dead chick embryo.

To study the effect of the status of p53 on the DNA damage response to SFOM-0046, we assessed the activation of markers of the DNA damage checkpoints (ATR, ATM, Chk1, γ-H2AX) and HR in isogenic HCT116 (p53 +/+ and p53 −/−) cancer cell lines. As shown in Fig. 7, treatment with SFOM-0046 for 24 h activated ATR and ATM pathways in both cell lines at all concentration tested. In addition, the treatment of the cells with either 1.5 or 3 μM of SFOM-0046 induced γ-H2AX and RAD51 foci in both cell lines indicating that SFOM-0046 induces also replication stress and DNA DSBs that are repaired by HR (Fig. 8A–D). These results show that p53 has relatively minor effects on the early stages of the DNA damage response induced by SFOM-0046 and that the DNA damage response to SFOM-0046 is a general mechanism and is not cancer cell line specific. Nonetheless, the role of p53 to trigger pro-survival biological processes is evidenced by the result showing that p53-deficient cancer cells are more sensitive to the drug than p53-proficient cancer cells.

### SFOM-0046 exhibits dose-dependent antitumor activity in the CAM Assay

We investigated the dose-response effect of SFOM-0046 on HT-1080 by using the CAM assay. To that end, cisplatin (10 μg per egg) was used as positive control and showed that the size of the tumours decreases by 50% as compared to untreated controls without increasing the chick embryos mortality (5%). Our excipient (Cremophor^®^ EL/99% ethanol/PBS, 1/1/14 v/v) did not affect tumour growth but slightly increased the embryos lethality (from 6% to 10%, Fig. 8E). When treated with 3 μg of SFOM-0046 per egg, the tumour sizes decreased to 85% of the control tumours. The tumour size dropped to 82% of the controls at 10 μg of SFOM-0046, and 53% at 30 μg/egg reaching the tumour size obtained for the positive control. SFOM-0046 showed also very low toxicity on the chick embryos. Embryos mortality was less or identical to the one caused by the excipient alone showing 10%, 7% and 9% for 3, 10 and 30 μg/egg of SFOM-0046, respectively. These results show for the first time that the inhibition of the growth of HT-1080 tumours by SFOM-0046 is dose-dependent.

## Discussion

Drugs that impact on DNA replication are amongst the most useful anticancer agents used in clinic^32^,^33^,^34^. We previously reported the development of new compounds showing antiproliferative activity^22^. Among them, the prototypical SFOM-0046 emerged as attractive for development due to its simple molecular structure, its antiproliferative activity in the low micromolar range against HT29, M21, and MCF-7 cell lines, its ability to block the cell cycle progression in S-phase on Jurkat cells, its ability to induce DNA replication stress (induction of γ-H2AX on M21 cells) showing DNA damage and its significant antitumour activity at 30 μg/eggs with low toxicity in the CAM assay^22^. The molecular mechanism underlying the generation of DNA damages by SFOM-0046 leading to an accumulation of cancer cells in S-phase is not completely deciphered yet. However, the SFOM-046 molecular structure excludes the possibility of direct DNA interaction mechanisms involving either alkylation or DNA intercalation. Indeed, the latter mechanisms are attributed to molecular structures that generate electrophilic species, unstable leaving groups or planar structures. The molecular structure of SFOM-0046 bearing an ethylurea substituent on the aromatic ring A, an ethyl group on aromatic ring B, and a sulfonate bridge between the two aromatic rings does not meet these criteria. In this study, we further investigated the DNA replication stress induced by SFOM-0046 and the pathways involved in the blocking of cancer cells in the S-phase.

Our study confirms that the SFOM-0046-induced cell cycle arrest and DNA replication stress are not cell-line specific. This was confirmed in M21, HT29, HT-1080 and HeLa cells and corroborated by our results obtained using Jurkat cells in the screening of PUB-SOs and the selection of SFOM-0046 as our prototypical PUB-SOs^22^. When studying the DNA damage response induced by SFOM-0046 in M21 and HT29 cells after a 24-h treatment, we observed that the drug induces preferentially the ATR-Chk1 pathway at all the concentrations tested while the ATM-Chk2 pathway was only weakly activated. Then, time-course activation of ATR and Chk1 in response to SFOM-0046 treatment (6 μM) revealed that ATR was quickly activated (2 h) followed by a delayed, strong activation of Chk1 (6 h). As known, ATR was activated in response to persistent single-stranded DNA breaks that occur when the replication forks stall and the replicative helicases continue to unwind the DNA ahead of the replication fork^35^. ATR-Chk1 plays an important role in the activation of the intra-S-phase checkpoints in response to DNA replication stress during normal cell division. In addition, it is a main mediator of G2/M checkpoints to prevent the presence of DNA damage during mitosis^36^. The rapid and selective activation of ATR after a short period in presence of SFOM-0046 suggests that the accumulation of cells in the S-phase is initially a consequence of the activation of the ATR-Chk1 pathway. This is also supported by a much higher induction of phosphorylated Chk1 than Chk2 in M21 and HT29 cells treated with SFOM-0046. However, the activation of the ATR-Chk1 pathway is not the only mechanism involved in the S-phase arrest. Indeed, the accumulation of cells in the S-phase begins to be observed at 6 h (Fig. S3), several hours after the beginning of the activation of the ATR-Chk1 pathway. Nonetheless, the activation of both the ATR-Chk1 and ATM-Chk2 pathways suggests that the two pathways may be involved in the activation of the intra-S-phase checkpoints and it also supports the work on ionising radiation and topoisomerase inhibitors showing that persistent activation of the intra-S-phase checkpoint leads to an accumulation of cells in the S-phase^36^,^37^.

Next, we characterized the type of DNA damage induced by SFOM-0046. Besides the fact that the phosphorylation of H2AX into γ-H2AX is considered as a hallmark of the number of DSBs generated, it has also been reported to be activated by other stresses including changes in the chromatin structure. Based on this, we confirmed that SFOM-0046 induces DNA DSBs, using 53BP1 as additional marker of DSBs. 53BP1 is actually known to be an important regulator of the DSB repair pathway choice that preserves DSB ends and antagonizes their resection. According to current models, DSBs are channelled into a particular DNA repair pathway via regulation of 53BP1 recruitment or inhibition at the break sites depending on the state of the cells^38^. In G1 phase, 53BP1 promotes NHEJ and prevents HR while its inhibition in S-phase contributes to promote HR by mechanisms still under investigation, but that probably involve several proteins including BRCA1^28^. By assessing DNA-PKcs and RAD51 foci, known as specific markers of NHEJ and HR repair respectively, we found that the SFOM-0046-induced DSBs are repaired by HR rather than NHEJ. This result strongly suggests that new inhibitors of the HR pathway will sensitize the anticancer activity of SFOM-0046.

Subsequently, we observed that M21 cells were more sensitive to SFOM-0046 than HT29 cells. Interestingly, the expression and activation of p53 in M21 cells are impaired compared to HT29 cells. p53 is a key protein that acts as a tumor suppressor and responds to diverse cellular stresses including DNA damage, oncogene activation or hypoxia. It orchestrates and modulates several cellular processes such as apoptosis, cell-cycle arrest, senescence and DNA repair in response to DNA damage^39^. Using isogenic HCT116 p53+/+ and p53−/−, we first confirmed that p53−/− cells are more sensitive to SFOM-0046 treatment than p53+/+ cells. We also confirmed that both isogenic HCT116 cell lines activate ATR and HR in a very similar way in response to DNA DSBs induced by SFOM-0046. Interestingly, SFOM-0046 activates more strongly ATM in HCT116 cells than in HT29 and M21 cells. This suggests that the activation of ATM is cell-line dependent and the increased survival of p53-proficient cells is not related to an increase or a change in DNA damage response but to another p53-dependent cellular process. Furthermore, we performed *in vivo* assays on human fibrosarcoma HT-1080 tumours using CAM assay. First, we confirmed that SFOM-0046 acts through a mechanism of action causing cell cycle arrest in S-phase in HT-1080 cells. Second, we found that the antitumor activity of SFOM-0046 in the CAM-assay is dose-dependent. However, based on relatively low solubility the maximal tolerated dose and the maximal antitumor activity of SFOM-0046 were not reached. Nevertheless, SFOM-0046 appears as a promising new anticancer agent. The mechanism of action, the antitumor activity and the toxicity of SFOM-0046 in mammal models will be further investigated to determine the potential of this new class of anticancer agents in a clinical setting.

## Methods

### Reagents and chemicals

SFOM-0046 was dissolved in DMSO at 40 mM and cisplatin was dissolved in water at 2 mM. To avoid cytotoxicity, the final concentration of DMSO in the culture medium was maintained under 0.2% (v/v). SFOM-0046 was prepared as published previously^22^. Cisplatin and DMSO were purchased from Sigma/Aldrich Chemicals (St-Louis, MI) and used as received. The antibodies pATM (Ser1981), pp53 (Ser15), pChk1 (Ser317) and pChk2 (Thr68) were purchased from Cell Signalling Technology (New England Biolabs ltd, ON). Anti-pATR (Thr1989) was obtained from Genetex (Irvine, CA), anti-phospho histone H2AX from Millipore (Ser139, clone JBW301, Billerica, MA) and anti-RAD51 from Santa Cruz (Dallas, TX). Anti-pDNA-PKcs (Thr2609) and anti-p53 were purchased from Abcam, and anti-53BP1 from Novus. All other chemicals were purchased from Bio-Rad (Hercules, CA), Roche Diagnostic Corporation (Indianapolis, IN) and Sigma/Aldrich Chemicals (St-Louis, MI).

### Cells and cell culture

Human skin melanoma M21 cells were provided by Dr. David A. Cheresh (U. of California in San Diego, CA). HT29, HT-1080 and HeLa cells were purchased from the American Type Culture Collection (Manassas, VA). Cell lines were maintained at 37 °C in a moisture-saturated atmosphere containing 5% of CO_2_ and were cultured in DMEM containing NaHCO_3_ (2.2 g/L), glucose (4.5 g/L) and glutamine (292 μg/mL), supplemented with 5% Fetal Bovine Serum (FBS) (Hyclone, Utah). Isogenic HCT116 p53+/+ and HCT116 p53−/− cells (kindly provided by Dr. Bert Vogelstein, Johns Hopkins University) were cultured in McCoy’s 5A medium, supplemented with 10% FBS, 1% penicillin-streptomycin.

### Cell cycle analysis

M21, HT29, HT-1080 and HeLa cells were seeded at 2 × 10^5^ cells per well in 6-well plates overnight and incubated with escalating concentrations of either SFOM-0046 or cisplatin for 24 h. DMSO was used as control. Cells were trypsinized, floating and adherent cells were pooled and washed with PBS, resuspended in 250 μL of PBS and fixed by addition of 750 μL of ice-cold anhydrous ethanol. Cells were centrifuged for 5 min and pellets were resuspended in PBS containing (2 μg/mL) DAPI. Cell cycle distribution was analysed using an Epics ESP flow cytometer (Coulter Corporation, Miami, FL).

### Fluorescence microscopy

Cells were seeded at 1.5 × 10^5^ cells (M21 and HT29) or 3 × 10^5^ cells (HCT116) per well in 6-well plates containing glass coverslips. The next day, cells were treated with SFOM-0046 at concentrations ranging from 1.5 to 6 μM or 30 μM of cisplatin for 24 h. DMSO was used as negative control. For RAD51 and γ-H2AX detection, cells were first washed in PBS, incubated in cytoskeleton buffer (10 mM PIPES pH 6.8, 100 mM NaCl, 300 mM sucrose, 3 mM MgCl_2_, 1 mM EGTA, 0.5% Triton X-100) for 5 min on ice, followed by incubation in cytoskeleton stripping buffer (10 mM Tris-HCl at pH 7.4, 10 mM NaCl, 3 mM MgCl_2_, 1% Tween 40, 0.5% sodium deoxycholate) for 5 min on ice. After 2 washes with ice-cold PBS, cells were fixed for 20 min in 4% paraformaldehyde in PBS and permeabilized in 0.5% Triton X-100 for 15 min at room temperature. Cells were blocked with PBS-10% goat serum for 1 h and incubated with anti-RAD51 (1:400) and anti-γ-H2AX (1:5000) primary antibodies for 2 h at room temperature. Coverslips were washed twice in PBS for 5 min, incubated with the secondary antibodies for 1 h at room temperature and washed again as above. For 53BP1 and pDNA-PKcs staining, cells were fixed with 4% paraformaldehyde in PBS for 10 min, washed with TBS and fixed with cold methanol (−20 °C) for 5 min. Next, cells were permeabilized with PBS containing 0.2% Triton X-100 for 5 min and washed three times 5 min with TBS. Then, cells were quenched with 0.1% sodium borohydride for 5 min, washed once with TBS, blocked in PBS containing 10% goat serum and 1% BSA for 1 h and incubated with the anti-53BP1 (1:500) and anti-pDNA-PKcs (Thr2609, 1:500) primary antibodies diluted in PBS 1% BSA for 2 h at room temperature. Coverslips were washed three times 10 min with TBS prior to a 1-h incubation with the appropriate secondary antibody to be rinsed again as above. In each case, Alexa Fluor 488 and/or Alexa Fluor 568 (Invitrogen) were used as secondary antibodies (1:1000). Coverslips were mounted onto slides with PBS-glycerol (90%) containing 1 mg/ml paraphenylenediamine and 0.2 mg/ml of 4, 6-diamidino-2-phenylindole (DAPI).

For each condition, at least 100 cells from three independent trials were scored for γ-H2AX, RAD51, 53BP1, and pDNA-PKcs foci formation. For foci scoring, images were acquired using a Leica CTR 6000 microscope. Then, the number of foci per cell was automatically counted according to intensity following background subtraction and deconvolution using Volocity software v 6.0 (Perkin-Elmer Improvision).

### Western blot analysis

M21, HT29, HT-1080 and HCT116 (p53+/+ and p53−/−) cells were seeded in 60 mm petri dishes at 7 × 10^5^ cells per dish and incubated overnight. Cells were incubated with escalating concentrations of SFOM-0046 or cisplatin for 24 h. DMSO was used as a control. Thereafter, cells were trypsinized, collected and washed with PBS. Then, cells were resuspended in lysis buffer (50 mM Tris-HCl, pH 7.5, 500 mM NaCl, 0.5% NP-40) containing protease and phosphatase inhibitors (PMSF (1 mM), aprotinin (0.019 TIU/ml), leupeptin (1 μg/ml), NaF (5 mM) and Na_3_VO_4_ (1 mM)), incubated for 30 min on ice, and lysed by sonication. Insoluble material was removed by high-speed centrifugation at 4 °C and the protein concentration was determined by the Bradford assay. Forty micrograms of proteins were subjected to electrophoresis using 10% bisacrylamide gels or NuPage 3–8% Tris-acetate gels (Novex, Invitrogen). Proteins were transferred onto nitrocellulose membranes and incubated with 5% (w/v) non-fat dry milk in 1X TBST (TBS, pH 7.4 and 0.1% Tween-20™) for 1 h at room temperature and then with antibodies pATR (Thr1989, 1:1000), pATM (Ser1981, 1:1000), pp53 (Ser15, 1:1000), p53 (1:1000), pChk1 (Ser317, 1:1000), pChk2 (Thr68, 1:1000). Membranes were washed with TBST and incubated in presence of 1:5000 peroxidase-conjugated immunoglobulins in TBST for 1 h at room temperature. After washing the membranes with TBST, detection of the immunoblot was carried out using an enhanced chemiluminescence (ECL) detection reagent kit. Anti β-actin-HRP (1:10000) (Santa Cruz Biotechnology, CA) was used as control of the protein amount on the gels.

### MTT assay

M21 and HT29 cells were seeded in 96-well microtiter plates (Thermo Scientific™ Nunc™ MicroWell™ 96-Well Optical-Bottom Plates with Polymer Base) at 1 × 10^3^ and 4 × 10^3^ cells per well, respectively, in 100 μL of medium. Plates were incubated for 24 h. Medium was replaced with medium containing escalating concentrations of drugs and the plates were incubated for 96 h. Afterwards, 10 μL of 3-(4,5-Dimethyl-2-thiazolyl)-2,5-diphenyl-2H-tetrazolium bromide (Thiazol Blue Tetrazolium Bromide 98% - M2128 - SIGMA) (5 mg/mL in water) were added to the wells. Four hours later, 100 μL of the solubilisation solution (10% sodium dodecyl sulfate (SDS) in 0.01 M HCl) was added and the precipitates were allowed to solubilize overnight at room temperature in the dark. The optical density was read using an Infinite^®^ F50/Robotic - Absorbance microplate readers (TECAN)) at 550 nM. The experiments were performed three times in triplicate.

### Viability of HCT116 cells

Cells were trypsinized from stock cultures and seeded into black-sided, clear bottom 96-well microplates (Corning, cat# 3603) at a concentration of 1 x 10^3^ cells per well in a volume of 100 μL. The following day, the media was replaced with the intended drug concentrations diluted in media. Cells were exposed to drug treatment for 96 h, then stained with Hoechst 33342 at 10 μg/mL in media for 30 min, at 37 °C, prior to imaging. Cells were imaged using a Cytation 5 Cell Imaging Multi-Mode Reader (BioTek Instruments). All cell counting experiments were performed at least four times in quadriplicates using a 4× microscope objective. Threshold was set to 10,000 with a minimum object size of 5 μm and a maximum size set to 50 μm. Data are presented as mean percent cell viability relative to control DMSO-treated cells ± S.D.

### CAM Assay

Freshly fertilized chicken eggs purchased from Couvoirs Victoriaville (Victoriaville, Quebec, Canada) were incubated for 10 days in a Pro-FI egg incubator (Lyon Electric, Chula Vista, CA) fitted with an automatic egg turner before being transferred to a Roll-X static incubator for the rest of the incubation period. Eggs were kept at 37 °C in a 60% relative humidity atmosphere for the entire incubation period. On day-10, a hole was drilled on the side of the egg using a hobby drill (Dremel, Racine, WI), and a negative pressure was applied to create a new air sac. A window was opened in that new air sac and was covered with transparent adhesive tape to prevent contamination. A freshly prepared HT-1080 cell suspension (40 μL, 3.5 × 10^5^ cells/egg) was applied directly on the freshly exposed CAM tissue. On day-11, drugs were extemporaneously diluted at the required concentrations in the excipient (Cremophor^®^ EL/99% ethanol/PBS, 1/1/14 v/v). The drug solution (100 μL) was injected into a vein under the CAM. Each experimental group contained 10–12 eggs that were incubated until day-17. Embryos were then sacrificed by cooling at 4 °C for at least 4 h. Tumours were collected, and tumour wet-weight were recorded. The number of dead embryos and signs of toxicity from the different groups were also recorded.

## Additional Information

**How to cite this article**: Pauty, J. *et al*. Investigation of the DNA damage response to SFOM-0046, a new small-molecule drug inducing DNA double-strand breaks. *Sci. Rep.* **6**, 23302; doi: 10.1038/srep23302 (2016).

## Supplementary Material

Supplementary Information

## Figures and Tables

**Figure 1:**
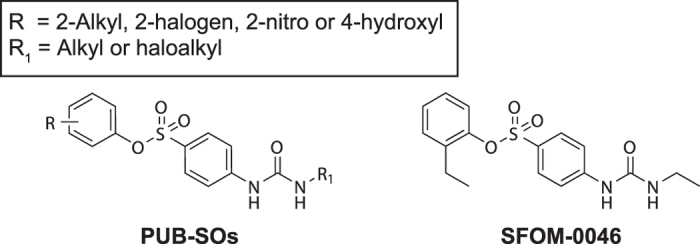
Chemical structure of PUB-SOs and SFOM-0046.

## References

[b1] C. Friedberg E., C. Walker G. & Siede W. DNA repair and mutagenesis. ASM Press, Washington, DC (1995).

[b2] Zhou B. B. & Elledge S. J. The DNA damage response: putting checkpoints in perspective. Nature 408, 433–439 (2000). 11100718 10.1038/35044005

[b3] Matsuoka S. . ATM and ATR substrate analysis reveals extensive protein networks responsive to DNA damage. Science 316, 1160–1166 (2007). 17525332 10.1126/science.1140321

[b4] Chen P. . The 1.7 A crystal structure of human cell cycle checkpoint kinase Chk1: implications for Chk1 regulation. Cell 100, 681–692 (2000). 10761933 10.1016/s0092-8674(00)80704-7

[b5] Matsuoka S., Huang M. & Elledge S. J. Linkage of ATM to cell cycle regulation by the Chk2 protein kinase. Science 282, 1893–1897 (1998). 9836640 10.1126/science.282.5395.1893

[b6] Bonner W. M. . YH2AX and cancer. Nat. Rev. Cancer 8, 957–967 (2008). 19005492 10.1038/nrc2523 PMC3094856

[b7] Culligan K. M., Robertson C. E., Foreman J., Doerner P. & Britt A. B. ATR and ATM play both distinct and additive roles in response to ionizing radiation. Plant J. 48, 947–961 (2006). 17227549 10.1111/j.1365-313X.2006.02931.x

[b8] Marechal A. & Zou L. DNA damage sensing by the ATM and ATR kinases. Cold Spring Harb. Perspect. Biol. 5, a012716 (2013). 24003211 10.1101/cshperspect.a012716 PMC3753707

[b9] Zimmermann M. & de Lange T. 53BP1: pro choice in DNA repair. Trends Cell Biol. 24, 108–117 (2014). 24094932 10.1016/j.tcb.2013.09.003 PMC3946699

[b10] Jackson S. P. Sensing and repairing DNA double-strand breaks. Carcinogenesis 23, 687–696 (2002). 12016139 10.1093/carcin/23.5.687

[b11] Mari P. O. . Dynamic assembly of end-joining complexes requires interaction between Ku70/80 and XRCC4. Proc. Natl. Acad. Sci. USA. 103, 18597–18602 (2006). 17124166 10.1073/pnas.0609061103 PMC1693708

[b12] Davis A. J. & Chen D. J. DNA double strand break repair via non-homologous end-joining. Transl. Cancer Res. 2, 130–143 (2013). 24000320 10.3978/j.issn.2218-676X.2013.04.02 PMC3758668

[b13] Chan D. W. . Autophosphorylation of the DNA-dependent protein kinase catalytic subunit is required for rejoining of DNA double-strand breaks. Genes Dev. 16, 2333–2338 (2002). 12231622 10.1101/gad.1015202 PMC187438

[b14] Ma Y., Pannicke U., Schwarz K. & Lieber M. R. Hairpin opening and overhang processing by an Artemis/DNA-dependent protein kinase complex in nonhomologous end joining and V(D)J recombination. Cell 108, 781–794 (2002). 11955432 10.1016/s0092-8674(02)00671-2

[b15] Wilson T. E., Grawunder U. & Lieber M. R. Yeast DNA ligase IV mediates non-homologous DNA end joining. Nature 388, 495–498 (1997). 9242411 10.1038/41365

[b16] Stracker T. H. & Petrini J. H. The MRE11 complex: starting from the ends. Nat. Rev. Mol. Cell Biol. 12, 90–103 (2011). 21252998 10.1038/nrm3047 PMC3905242

[b17] Suwaki N., Klare K. & Tarsounas M. RAD51 paralogs: roles in DNA damage signalling, recombinational repair and tumorigenesis. Semin. Cell Dev. Biol. 22, 898–905 (2011). 21821141 10.1016/j.semcdb.2011.07.019

[b18] Buisson R. . Cooperation of breast cancer proteins PALB2 and piccolo BRCA2 in stimulating homologous recombination. Nat. Struct. Mol. Biol. 17, 1247–1254 (2010). 20871615 10.1038/nsmb.1915 PMC4094107

[b19] Krejci L., Altmannova V., Spirek M. & Zhao X. Homologous recombination and its regulation. Nucleic Acids Res. 40, 5795–5818 (2012). 22467216 10.1093/nar/gks270 PMC3401455

[b20] Symington L. S. & Gautier J. Double-strand break end resection and repair pathway choice. Annu. Rev. Genet. 45, 247–271 (2011). 21910633 10.1146/annurev-genet-110410-132435

[b21] Gagne-Boulet M. . Synthesis and biological evaluation of novel *N*-phenyl ureidobenzenesulfonate derivatives as potential anticancer agents. Part 2. Modulation of the ring B. Eur. J. Med. Chem. 103, 563–573 (2015). 26408815 10.1016/j.ejmech.2015.09.012

[b22] Turcotte V. . Synthesis, biological evaluation, and structure-activity relationships of novel substituted *N*-phenyl ureidobenzenesulfonate derivatives blocking cell cycle progression in S-phase and inducing DNA double-strand breaks. J. Med. Chem. 55, 6194–6208 (2012). 22694057 10.1021/jm3006492 PMC3395254

[b23] Siddik Z. H. Cisplatin: mode of cytotoxic action and molecular basis of resistance. Oncogene 22, 7265–7279 (2003). 14576837 10.1038/sj.onc.1206933

[b24] Rogakou E. P., Nieves-Neira W., Boon C., Pommier Y. & Bonner W. M. Initiation of DNA fragmentation during apoptosis induces phosphorylation of H2AX histone at serine 139. J. Biol. Chem. 275, 9390–9395 (2000). 10734083 10.1074/jbc.275.13.9390

[b25] Rogakou E. P., Boon C., Redon C. & Bonner W. M. Megabase chromatin domains involved in DNA double-strand breaks *in vivo*. J. Cell Biol. 146, 905–916 (1999). 10477747 10.1083/jcb.146.5.905 PMC2169482

[b26] Sancar A., Lindsey-Boltz L. A., Unsal-Kacmaz K. & Linn S. Molecular mechanisms of mammalian DNA repair and the DNA damage checkpoints. Annu. Rev. Biochem. 73, 39–85 (2004). 15189136 10.1146/annurev.biochem.73.011303.073723

[b27] Wang H. . A perspective on chromosomal double strand break markers in mammalian cells. Jacobs J. Radiat. Oncol. 1, 1–8 (2014). PMC4299656 25614903

[b28] Chapman J. R., Taylor M. R. & Boulton S. J. Playing the end game: DNA double-strand break repair pathway choice. Mol. Cell 47, 497–510 (2012). 22920291 10.1016/j.molcel.2012.07.029

[b29] Reddy Y. V., Ding Q., Lees-Miller S. P., Meek K. & Ramsden D. A. Non-homologous end joining requires that the DNA-PK complex undergo an autophosphorylation-dependent rearrangement at DNA ends. J. Biol. Chem. 279, 39408–39413 (2004). 15258142 10.1074/jbc.M406432200

[b30] Smith S. D. . Protein kinase Calpha (PKCalpha) regulates p53 localization and melanoma cell survival downstream of integrin alphav in three-dimensional collagen and *in vivo*. J. Biol. Chem. 287, 29336–29347 (2012). 22773839 10.1074/jbc.M112.341917 PMC3436133

[b31] Bao W. & Stromblad S. Integrin αv-mediated inactivation of p53 controls a MEK1-dependent melanoma cell survival pathway in three-dimensional collagen. J. Cell Biol. 167, 745–756 (2004). 15557124 10.1083/jcb.200404018 PMC2172581

[b32] Rahman A. M., Yusuf S. W. & Ewer M. S. Anthracycline-induced cardiotoxicity and the cardiac-sparing effect of liposomal formulation. Int. J. Nanomedicine 2, 567–583 (2007). 18203425 PMC2676818

[b33] Florea A. M. & Busselberg D. Cisplatin as an anti-tumor drug: cellular mechanisms of activity, drug resistance and induced side effects. Cancers 3, 1351–1371 (2011). 24212665 10.3390/cancers3011351 PMC3756417

[b34] Yount G., Yang Y., Wong B., Wang H. J. & Yang L. X. A novel camptothecin analog with enhanced antitumor activity. Anticancer Res. 27, 3173–3178 (2007). 17970058

[b35] Byun T. S., Pacek M., Yee M. C., Walter J. C. & Cimprich K. A. Functional uncoupling of MCM helicase and DNA polymerase activities activates the ATR-dependent checkpoint. Genes Dev. 19, 1040–1052 (2005). 15833913 10.1101/gad.1301205 PMC1091739

[b36] Xiao Z. . Chk1 mediates S and G2 arrests through Cdc25A degradation in response to DNA-damaging agents. J. Biol. Chem. 278, 21767–21773 (2003). 12676925 10.1074/jbc.M300229200

[b37] Falck J., Mailand N., Syljuasen R. G., Bartek J. & Lukas J. The ATM-Chk2-Cdc25A checkpoint pathway guards against radioresistant DNA synthesis. Nature 410, 842–847 (2001). 11298456 10.1038/35071124

[b38] Panier S. & Boulton S. J. Double-strand break repair: 53BP1 comes into focus. Nat. Rev. Mol. Cell Biol. 15, 7–18 (2014). 24326623 10.1038/nrm3719

[b39] Green D. R. & Kroemer G. Cytoplasmic functions of the tumour suppressor p53. Nature 458, 1127–1130 (2009). 19407794 10.1038/nature07986 PMC2814168

