# Supplementary material for: Investigation of the DNA damage response to SFOM-0046, a new small-molecule drug inducing DNA double-strand breaks
Source: Sci Rep. 2016 Mar 22;6:23302. doi: 10.1038/srep23302 (PMC4802344; doi:10.1038/srep23302)
Supplement: Supplementary Information [file srep23302-s1.pdf]

# Investigation of the DNA damage response to SFOM-0046, a new small-molecule drug inducing DNA double-strand breaks

Joris Pauty[1,2], Marie-France Côté[3], Amélie Rodrigue[1,2], Denis Velic[1,2], Jean-Yves Masson[1,2,4], Sébastien Fortin[3,5*]

[1] Genome Stability Laboratory, CHU de Quebec Research Center, Oncology Axis, Hôtel-Dieu-de-Québec, 9 McMahon, Quebec City, QC, G1R 2J6, Canada.

[2] Department of Molecular Biology, Medical Biochemistry and Pathology, Faculty of Medicine, Laval University, Quebec City, QC, G1V 0A6, Canada.

[3] CHU de Quebec Research Center, Oncology Axis, Hôpital Saint-François d'Assise, 10 de l'Espinay, Quebec City, QC, G1L 3L5, Canada.

[4] FRQS Chercheur National Investigator.

[5] Faculty of Pharmacy, Laval University, Quebec City, QC, G1V 0A6, Canada.

**Corresponding Author:** Sébastien Fortin, Faculty of Pharmacy, Laval University, Quebec City, QC, G1V 0A6, Canada. Phone: +1-418-525-4444 ext. 52364; Fax: +1-418-525-4372; E-mail: sebastien.fortin@pha.ulaval.ca

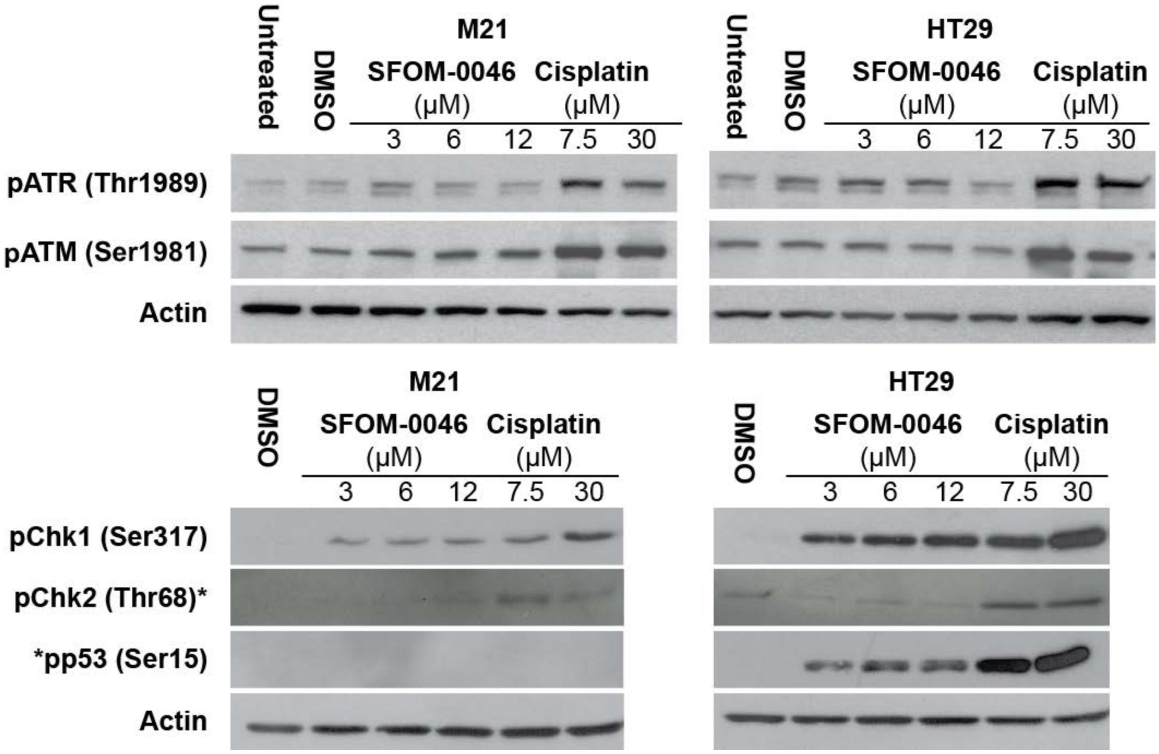


**Figure S1.** Full-length western blots of Figure 3.

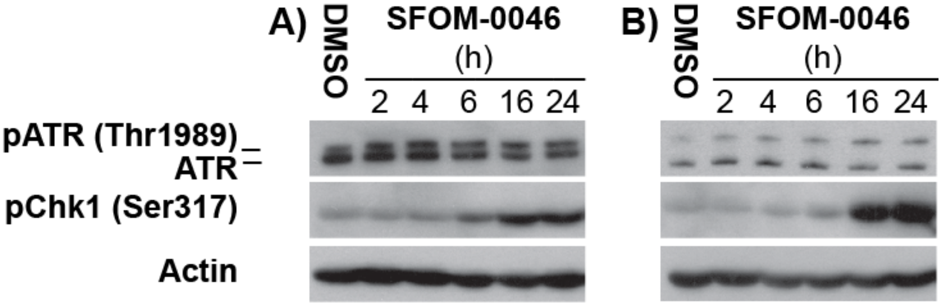


**Figure S2.** Effect of SFOM-0046 (6 μM) on the phosphorylation of ATR and Chk1 of A) M21 and B) HT29 cells after 2, 4, 6, 16 and 24 h of treatment.

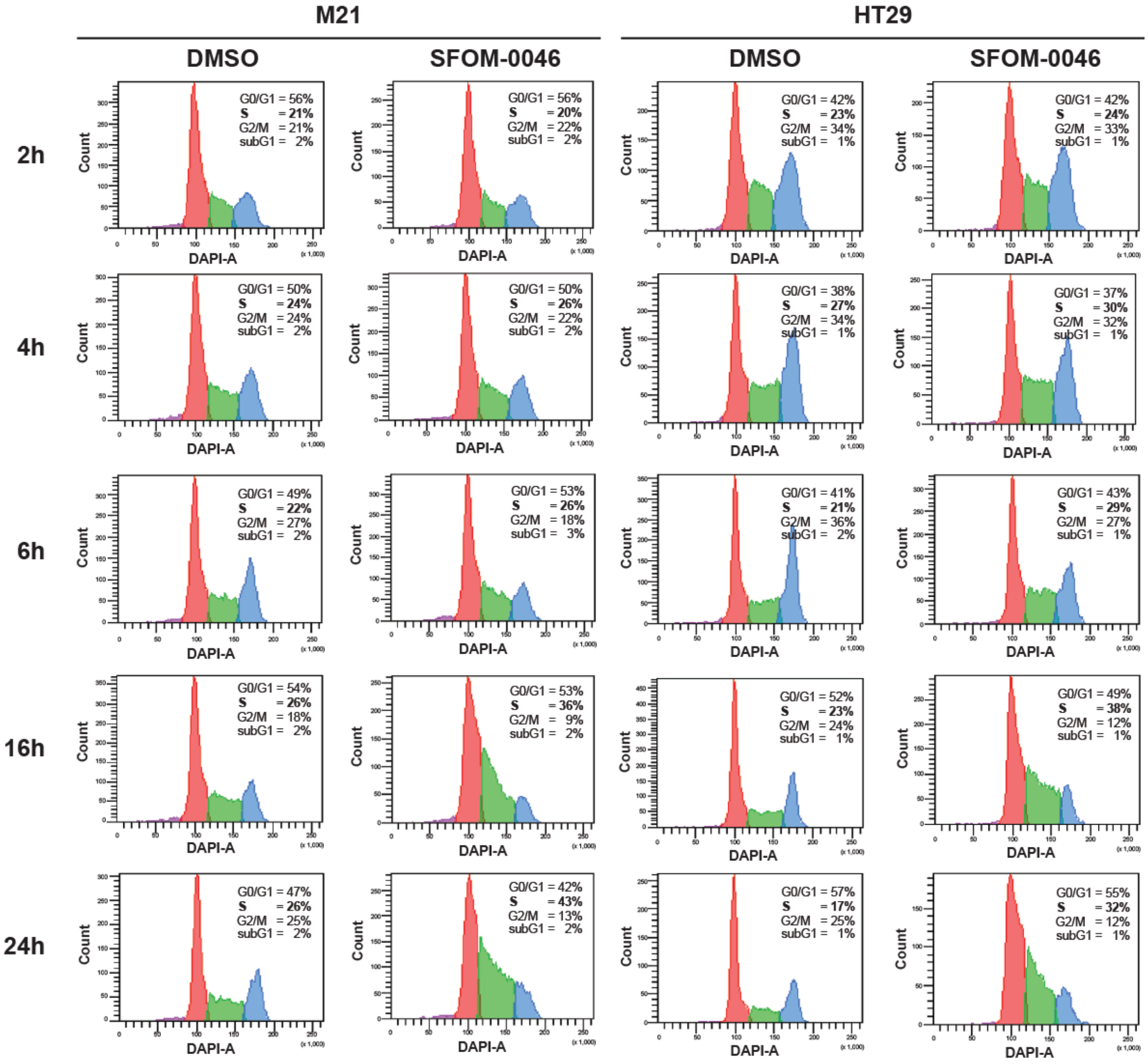


**Figure S3.** Effect on the cell cycle progression of M21 and HT29 cells of DMSO and SFOM-0046 (6 µM) after 2, 4, 6, 16 and 24 h of treatment.
